# Retrospective longitudinal assessment of optic nerve sheath diameter in patients with malignant glioma

**DOI:** 10.1002/cam4.6789

**Published:** 2023-12-08

**Authors:** Daniel Feucht, Susanne R. Kerscher, Christer Ruff, Martin U. Schuhmann, Constantin Roder, Julian Zipfel

**Affiliations:** ^1^ Department of Neurosurgery University Hospital of Tuebingen Tubingen Germany; ^2^ Department of Diagnostic and Interventional Radiology University Hospital of Ulm Ulm Germany; ^3^ Department of Diagnostic and Interventional Neuroradiology University Hospital of Tuebingen Tubingen Germany

**Keywords:** glioblastoma, intracranial pressure, optic nerve sheath diameter

## Abstract

**Introduction:**

Glioblastoma (GBM) is a tumor with rapid growth and a possible relationship to elevated intracranial pressure (ICP). High ICP may not always be associated with clinical signs. A non‐invasive technique for assessment of ICP is measuring the optic nerve sheath diameter (ONSD). Identifying patients who need immediate intervention is of importance in neuro‐oncological care. The goal of this study is to assess the available magnetic resonance imaging (MRI) of patients with GBM with respect to pre‐ and postoperative ONSD.

**Methods and Materials:**

Retrospective data analysis was performed on all patients operated for GBM at a tertiary care center between 2010 and 2020. Two pre and one postoperative MRI had to be available. Clinical data and ONSD at multiple time points were analyzed and correlated, as well as preoperative volumetrics.

**Results:**

Sixty‐seven patients met the inclusion criteria. Clinical signs of elevated ICP were seen in 25.4% (*n* = 17), while significant perifocal edema was present in 67.2% (*n* = 45) of patients. Clinical signs of preoperatively elevated ICP were associated with significantly elevated ONSD at diagnosis (*p* < 0.001) as well as preoperative tumor volume (*p* < 0.001). Significant perifocal edema at the time of diagnosis was associated with elevated ONSD (*p* = 0.029) and higher tumor volume (*p* = 0.003). In patients with significant edema, ONSD increased significantly between preoperative MRIs (*p* = 0.003/005). In patients with clinical signs of raised ICP, ONSD also increased, whereas it was stable in asymptomatic patients (yes: 5.01+/−4.17 to 5.83+/−0.55 mm, *p* = 0.010, no: 5.17+/−0.46 mm to 5.38+/−0.41 mm, *p* = 0.81). A significant increase of ONSD from diagnosis to preoperative MRI and a significant decrease until 3 months postoperatively were observed (*p* < 0.001).

**Conclusions:**

ONSD might help identify high ICP in patients with GBM. In this first‐of‐its kind study, we observed a significant increase of ONSD preoperatively, likely associated with edema. Postoperatively, ONSD decreased significantly until 3 months after surgery and increased again at 12 months. Further prospective data collection is warranted.

## Graphical abstract

Measurement of optical nerve sheath diameter (ONSD) is a noninvasive technique for assessment of intracranial pressure. Identifying patients at risk with the need of immediate intervention is of importance in neuro‐oncological care. This study retrospectively investigates pre‐ and postoperative ONSD in patients with glioblastoma.

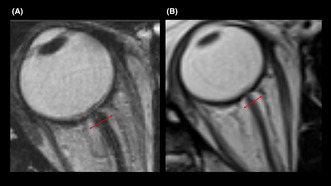

## 1 INTRODUCTION

Glioblastoma (GBM) is one of the highly malignant tumors of the central nervous system with rapid growth and association with elevated intracranial pressure (ICP). Intracranial hypertension may occur, among others, with progressive tumor volume, tumor‐associated perifocal edema, venous flow obstruction due to infiltration of draining blood vessels, or hydrocephalus.^1^ A general approximation of ICP via morphological imaging is not possible,^2^ but specific pathologies associated with elevated ICP such as hydrocephalus are accessible to CT or MR imaging. A technique for assessing ICP non‐invasively is measuring optic nerve sheath diameter (ONSD), both via ultrasound or specific MRI sequences.^3^ ONSD has been validated as a surrogate for ICP, shows good correlation with individual ICP dynamics,^4^, ^5^, ^6^, ^7^ and may correlate with outcome in TBI after hematoma removal.^8^ Data on ONSD in glioblastoma are scarce—in a heterogenous group of tumors, a decrease in ONSD was observed postoperatively.^9^

Suspicion of GBM requires timely surgery and concomitant adjuvant care. In the realities of patient care and limited resources, scheduling urgent intracranial surgery with regards to operating room capacity, optional intraoperative monitoring, postoperative intensive care monitoring, and inpatient capacity on the ward may be difficult. Recently, Muller et al.^10^ found that surgery within 1 month of diagnosis was reasonable in for most patients with GBM regarding extent of resection and outcome, while about 23% were operated on within the first 3 days. Identifying patients at risk due to elevated ICP with the need of immediate intervention as opposed to those in whom it would be reasonable to schedule surgery electively is important for patient management. Recent efforts have been undertaken to model glioma progression, local or generalized mass effect, and intracranial pressure to individual patient anatomy.^1^, ^11^ Still, no predictive variables have been established for decision‐making regarding urgent surgery.^12^

This study retrospectively assessed available MR imaging of patients with GBM with regards to pre‐ and postoperative ONSD and correlated them to clinical data in order to better identify patients at risk of raised ICP. The purpose of this study was to investigate the value of ONSD changes as a radiological marker of ICP in patients with GBM.

## 2 METHODS

All adult patients treated at our neurosurgical institution between 2010 and 2020 with the diagnosis of a GBM were included. We identified 749 cases in total. Main inclusion criteria for further analysis were: microsurgical resection of contrast‐enhancing tumor, at least two preoperative MRI scans at least 7 days apart with sufficient image quality, and with measurable ONSD on axial T2‐weighted sequences. An adequate slice of the optic nerve (on axial sequences) had to be present for ONSD measurement.

**FIGURE 1:**
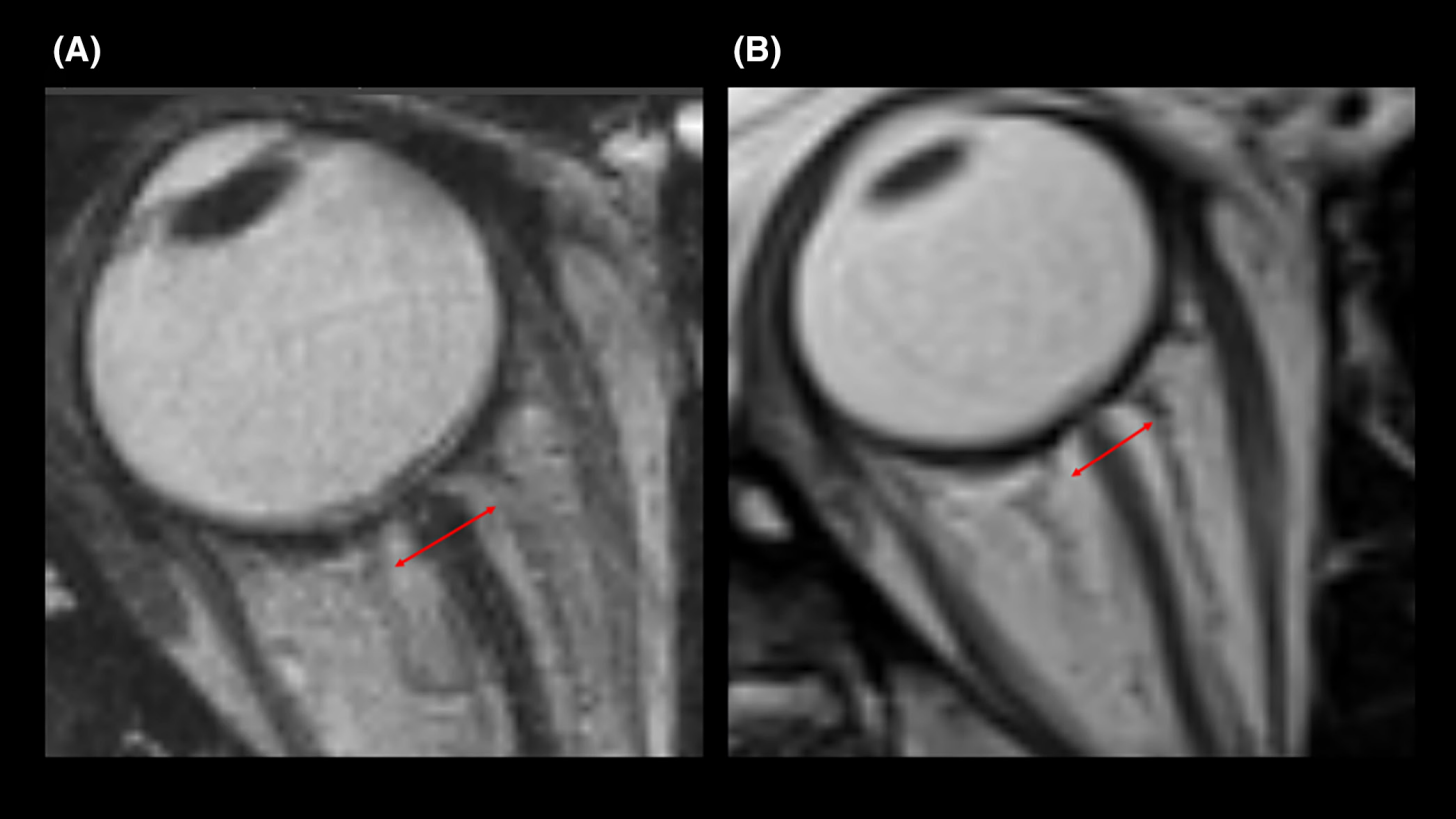
Exemplary (A) pre‐ and (B) postoperative MRI with ONSD marked red (6.4 and 5.8 mm); ONSD is measured 3 mm behind the optic disc perpendicular to the retina.

After meeting inclusion criteria, 67 patients were included for further analysis. We reviewed the clinical parameters, surgical data, as well as outcomes and all available imaging studies. Pre and postoperative MRI scans were assessed for ONSD as per protocol.^3^ Figure 1 gives an example of pre‐ and postoperative measurement of ONSD on MRI. Preoperative MRI was used to define tumor volume via volumetry as described before.^13^ Significant perifocal edema was defined as edema >1 cm in greatest diameter surrounding the tumor border and was evaluated on T2‐weighted images or fluid‐attenuated inversion recovery (FLAIR) sequences.^14^

We performed the statistical analysis using SPSS Statistics 28 (IBM). Continuous data were presented as mean (± standard deviation), whereas categorical data were shown as percentages. Continuous variables were tested for equality of variances by Levene's test. Normal distributed parametric variables with equal variances were compared using the unpaired or paired *t*‐test; otherwise, the Mann–Whitney *U* test was performed. Correlation of variables was tested via Pearson's or Spearman's correlation coefficient. Nominal variables were tested with the Fisher's exact test. A multivariate regression analysis was performed to investigate the influence of different independent parameters on the width of the ONSD. An F‐test was used to test the significance of the regression model. The effect size of the multivariate regression analysis was calculated using Cohen's effect size *f* ^2^. Here, *f* ^2^ = 0.02 corresponds to a weak effect, *f* ^2^ = 0.15 to a medium effect, and *f* ^2^ = 0.35 to a strong effect. *p* Values < 0.05 were regarded as significant. This study was performed in line with the principles of the Declaration of Helsinki. Institutional review board approval was granted by the Ethics Committee of University of Tuebingen (793/2022BO2) on December 21, 2022.

## 3 RESULTS

Among the 67 consecutive patients included, 29 were female (43.3%). Mean age at surgery was 60.8+/−11.9 a (22.7–88.2 a). Methylguanine methyltransferase (MGMT)‐promoter‐methylation status was available in all cases, and 35 tumors (52.2%) were unmethylated. Preoperative medication with dexamethasone was given in 37.3% (*n* = 25) with suspicion of increased ICP and/or marked perifocal edema on MRI. Mean follow‐up (FU) was 632+/−571.5 days, and 73.1% of patients died (*n* = 49) during the observation period. Clinical signs of elevated ICP (headaches, nausea, vomiting, impairment of consciousness) were seen in 25.4% (*n* = 17), and patients were dichotomized accordingly. Significant perifocal edema was present in 67.2% (*n* = 45) of patients.

Basic patient information, ONSD, tumor localization, adjuvant therapy regimens, and volumetric values are presented in Table 1. ONSD was measured at time of diagnosis (before administration of dexamethasone), preoperatively, and postoperatively (<72 h after surgery, 3 months, 12 months, last FU).

**TABLE 1:** Basic patient characteristics.

Age	Mean 60.8+/−11.9 years (range 22.7–88.2 years)								
Sex	43.3% Female (*n* = 29)				57.2% Male (*n* = 38)				
Follow‐up	Mean 632+/−572 days								
Localization	32.8% temporal (*n* = 22)	28.4% frontal (*n* = 19)	17.9% parietal (*n* = 12)	10.4% perirolandic (*n* = 7)	6.0% occipital (*n* = 4)		3.0% insula (*n* = 2)	1.5% diencephalo*n* (*n* = 1)	
MGMT Methylation status	52.2% unmethylated (*n* = 35)					47.8% methylated (*n* = 32)			
Clinical signs of raised ICP	No 25.4% (*n* = 17)					Yes 74,6% (*n* = 50)			
Significant edema	No 32.8% (*n* = 21)					Yes 67.2% (*n* = 45)			
Preoperative dexamethasone	No 62.8% (*n* = 42)					Yes 37.3% (*n* = 25)			
Preoperative tumor volume	Mean 27.65+/−24.92 (0.49–98.89) cm^3^								
Adjuvant therapy	52.2% Stupp. (*n* = 35)	19.4% radiotherapy alone (*n* = 13)	6.0% chemotherapy alone (*n* = 4)	4.5% GLARIUS (*n* = 3)	4.5% other studies (*n* = 3)	3.0% CETEG (*n* = 2)	1.5% Perry (*n* = 1)	1.5% EORTC (*n* = 1)	7.5% missing data (*n* = 5)

### 3.1 Clinical data

No influence of patients' age and sex on other clinical or radiological factors was identified. Mean initial ONSD in women was 5.24 ± 0.38 mm vs. 5.19 ± 0.57 mm, *p* > 0.05. A positive correlation was found between preoperative ONSD and need for dexamethasone administration (*p* = 0.019, Pearson = 0.353), signs of elevated ICP (*p* = 0.011, Pearson = 0.472), and preoperative tumor volume (*p* = 0.017, Pearson = 0.358). Postoperative ONSD correlated positively with signs of elevated ICP (*p* = 0.034, Pearson = 0.265).

Patients with preoperative dexamethasone medication had significantly higher ONSD preoperatively (*p* = 0.019, 5.34+/−0.37 vs. 5.66+/−0.49), concomitant with higher tumor volumes (*p* = 0.011, 21.74+/−22.0 vs. 37.58+/−26.82).

Significant perifocal edema at the time of diagnosis was associated with elevated ONSD (*p* = 0.029, 4.97+/−0.41 vs. 5.21+/−0.45) and higher tumor volume (*p* = 0.003, 15.96+/−23.57 vs. 33.37+/−23.76). Temporal as compared to parietal tumor localization was associated with lower ONSD values preoperatively at 3‐month and 12‐month FU (5.30+/−0.30 vs. 5.64+/−0.35 mm *p* = 0.16, 4.92+/−0.44 vs. 5.44+/−0.68 mm 0.23 and 4.94+/−0.56 vs. 5.56+/−0.69 mm *p* = 0.31, respectively). Tumor volumes were significantly larger in frontal (39.9+/−32.2 cm^3^) as compared to perirolandic (9.7+/−4.4 cm^2^, *p* = 0.022) temporal (18.7+/−18.6 cm^3^, *p* = 0.012) tumors. Parietal tumors (27.6+/−21.3 cm^3^) were significantly larger than perirolandic tumors (*p* = 0.044). Frontal tumors were associated with a higher likelihood of significant edema (89%) as compared to perirolandic (14%, *p* < 0.001), temporal (55% *p* = 0.007), and frontal GBM were associated with significant prevalence of clinical signs of raised ICP (42%), whereas no such symptoms were observed in perirolandic or parietal tumors.

Other localizations were not associated with significant differences in ONSD or other clinical parameters.

Clinical signs of elevated ICP preoperatively were associated with significantly elevated ONSD pre‐ and postoperatively (diagnosis: *p* < 0.001, 5.02+/−0.42 vs. 5.44+/−0.31; preoperatively: *p* = 0.006, 5.46+/−0.42 vs. 5.73+/−0.40; postoperatively: *p* = 0.017, 5.19+/−0.50 vs. 5.51+/−0.54) as well as tumor volume (*p* < 0.001, 20.61+/−18.3 vs. 48.37+/−30.40).

Unmethylated MGMT status was associated with a higher rate of death during FU (*p* = 0.015, Pearson = −0.297). No difference in ONSD was observed at any time points. No impact of sex on ONSD or tumor volume was observed.

An F‐test showed that the analysis of the effect of multiple independent parameters on ONSD was a significant regression model at *p* < 0.05. Multivariate regression analysis showed a significant influence of the presence of clinical ICP signs (*p* < 0.05) and perirolandic tumor location (*p* < 0.01) on initial ONSD with a regression coefficient B of 0.489 or 0.416, respectively. This means that the presence of clinical ICP signs or a perirolandic tumor localization increases the ONSD value by an average of 0.489 and 0.416 mm, respectively. All other tumor locations, age, sex, MGMT methylation status, tumor volume, and edema did not yield a significant regression coefficient B in the analysis (*p* > 0.05). The adjusted *R* ^2^ resulted in a value of 0.215. The effect size, according to Cohen, showed *f* ^2^ = 0.27 and thus a medium effect of the investigated independent parameters on the ONSD.

### 3.2 ONSD‐dynamics

**FIGURE 2:**
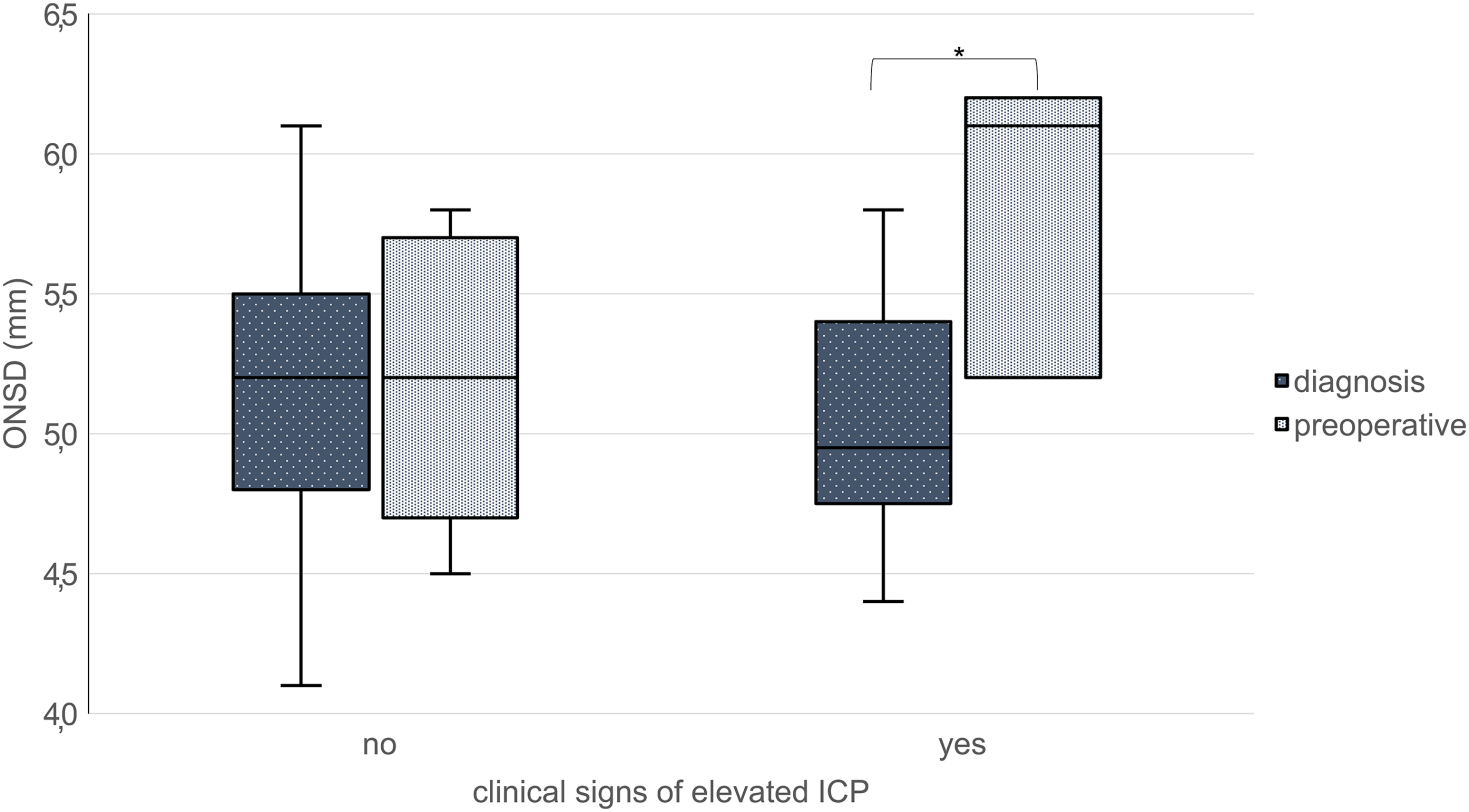
Boxplot of ONSD at time of diagnosis versus preoperatively in patients with and without clinical signs of elevated ICP *p < 0.05.

Analyzing the preoperative course of ONSD did not elicit any significant effect of MGMT status, sex, or dexamethasone medication, though the sample size was too small to properly evaluate the effect of the latter. In patients with significant edema in imaging, ONSD increased significantly between the MRIs at diagnosis and preoperatively in patients with and without edema (yes: 5.17+/−0.38–5.46+/−0.44 mm, *p* = 0.003; no: 4.82+/−0.38–5.26+/−0.31 mm, *p* = 0.005). In patients with clinical signs of raised ICP, ONSD also increased, whereas not in asymptomatic patients (yes: 5.01+/−4.17–5.83+/−0.55 mm, *p* = 0.010, no: 5.17+/−0.46 mm–5.38+/−0.41 mm, *p* = 0.81; See Figure 2). 37.3% (*n* = 25) of patients received dexamethasone after the initial scan. Subgroup analysis for dexamethasone medication yielded no significant differences. No other medications to alleviate ICP were administered. No differences in age and sex were observed between these groups. No impact of type of adjuvant therapy on ONSD was observed.

**FIGURE 3:**
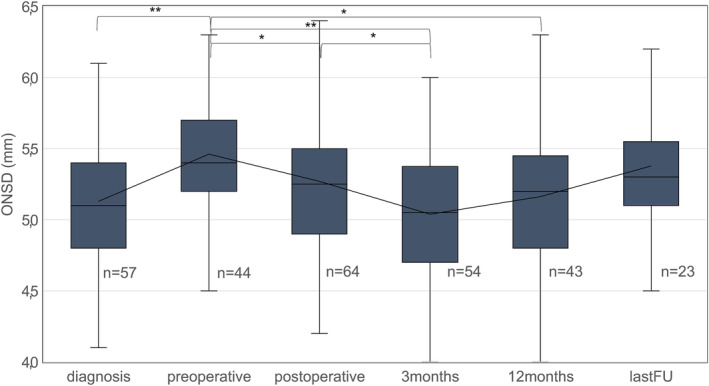
Boxplot of mean ONSD values from time of diagnosis until last FU *p < 0.05, **p < 0.001.

The chronological changes of ONSD are shown in Figure 3 and Table 2.

**TABLE 2:** Course of ONSD.

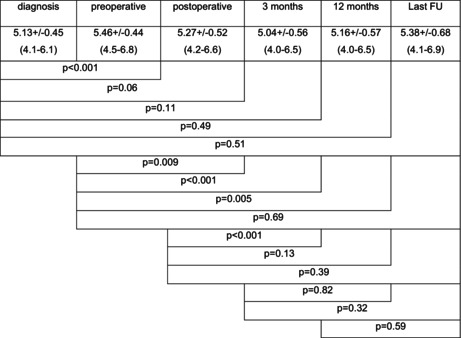

A significant increase of ONSD from initial diagnosis to the preoperative MRI and a significant decrease until 3 months postoperatively were observed. At 12 months postoperatively, ONSD was still lower than preoperatively. At last FU, there was no difference to preoperative values.

The mean decrease of ONSD from pre‐ to postoperative MRI was 0.17+/−0.42 mm (−0.7 to 0.9) and 0.33+/−0.34 mm (−0.6 to 1.0, *p* = 0.012) from preoperative to 3 months postoperative MRI.

## 4 DISCUSSION

Our results showed an increase in ONSD in between the time of diagnosis and the time of surgery among patients with newly diagnosed GBM. This was especially pronounced in patients with clinical signs of elevated ICP. Preoperative dexamethasone did not significantly impact ONSD. Due to small sample size in subgroup analysis, the statistical power of this observation is not high enough; further investigation is needed. The correlation of patients receiving dexamethasone correlating with edema, larger tumors, and clinical signs of elevated ICP might bias this observation.

After surgery and during adjuvant therapy, ONSD decreased significantly with the lowest values at 3 months postoperatively. At the last FU, ONSD had increased to preoperative values.

Previous data of postoperative decrease of ONSD was confirmed in our study.^9^ The effects of postoperative adjuvant therapy on the observed continuing decrease after surgery have to be further analyzed. Preoperative tumor‐associated and focal edema from intraoperative manipulation, which resolves over time, may also be considered as a cause of further ONSD decline during the course. Additionally, slow regression of ONSD may also be observed due to physiological processes after prolonged or severe ICP elevation in the sense of hysteresis.^15^

As an established surrogate of approximating ICP, we found that ONSD had a reasonable correlation (*r* = 0.472, *p* < 0.05) with clinical indices of intracranial hypertension. This agrees with a recent study of adult neurological patients with ICP elevation and invasive ICP monitoring. The correlation between ONSD measured on CT and ICP was *r* = 0.5, *p* < 0.05.^16^ Jiang et al.^17^ were able to demonstrate similar findings when correlating ONSD with cerebrospinal fluid pressure in patients with leptomeningeal metastasis. However, the non‐linearity of the relationship between ONSD and ICP has to be considered. Many inter‐individual (patho) physiological factors might influence the width of the optic nerve sheath, for example, type of pathology,^18^ duration of ICP increase,^19^ intraocular pressure,^20^, ^21^ communication between optic and intracranial subarachnoid spaces,^21^, ^22^ as well as sex and age.^23^ These individual factors may be significant confounders in our cohort with GBM: preoperatively, a satisfactory correlation between ONSD and ICP signs as well as tumor volume was found. A multivariate regression analysis showed a significant influence of central tumor location and the presence of clinical ICP signs on the width of the ONSD. The presence of these parameters leads on average to an increase in ONSD of 0.416 and 0.489 mm, respectively. In the multivariate analysis, the influence of the other parameters (age, sex, edema, tumor volume, all other tumor locations, and MGMT methylation status) studied was not significant. The adjusted R^2^ of 0.215 indicates that in this model, 21.5% of the scatter of the ONSD can be explained by the examined parameters with an overall medium effect size. This is consistent with the assumption that the width of the ONSD follows very individual, complex, partly not yet understood, dynamic processes and is influenced by numerous other factors that were not considered in our study, e.g., the extent and duration of untreated ICP elevation and the velocity of development of ICP increase.^4^ Patients with GBM may, moreover, experience high ICP due to tumor volume, venous outflow obstruction, or hydrocephalus, but we found a significant correlation especially with edema in agreement with current literature.^1^

Unsurprisingly, we found frontal tumors to be larger at diagnosis and associated with more edema as well as higher ONSD as compared to other localizations. Also frontal localization was associated with higher prevalence of clinical signs of raised ICP.

The influence of inter‐individual (patho) physiological factors on the width of optic nerve sheath elicits the fact that the informative power of ONSD regarding ICP can certainly be too low in a single measurement. Repeated measurements over longer periods of time are therefore necessary. The dynamics of the ONSD in relation to clinical symptoms and imaging findings may allow a more accurate and reliable statement about ICP.

An additional factor to consider is the lack of reference values for ONSD in adults. Korean data from 314 healthy adults yielded a mean MRI‐based ONSD of 4.71 mm. Sex and age had no significant effect on ONSD.^24^ Rohr et al.^25^ found a mean MRI‐based ONSD of 5.3 mm in 123 adults without evidence of increased ICP, also without influence of age. In our cohort, regarding the values in the 3 M FU examination as “normal values” in asymptomatic patients, we found a mean value of 5.0 mm.

To our knowledge, this is the first study to assess intracranial hypertension non‐invasively in patients with GBM. ONSD is a promising adjunct for patient care. With MRI‐based and sonographically measured ONSD being comparable,^3^, ^26^, ^27^ this tool might help assess an estimation of ICP without the need for multiple MRI scans. Thus, patients with GBM and urgent need for intervention due to assumed increased ICP might be identified early. The sonography‐based ONSD, on the other hand, may be an adjunct to follow‐up patients quickly and easily without MRI. Preoperative as well as 3‐month values might thus define the upper and lower limits of ONSD, respectively. Especially in the case of new symptoms or in the context of reducing corticosteroids, ONSD might be used to evaluate concerns for raised ICP without the need of MRI. Additionally, ONSD may be used to assess the effects of adjuvant therapy on ICP, and future studies should evaluate a correlation with oncological tumor control. However, ultrasound may systematically measure slightly larger ONSD values compared to MRI,^3^, ^28^ which needs to be considered when using both modalities.

In our clinical experience, elevated ICP may be difficult to assess especially in situations where patients have profound neurological deficits such as hemiparesis, aphasia, and cognitive impairment. These confounders may limit the significance of clinical examination. In these clinical contexts, therefore, ONSD might help identify patients at risk due to elevated ICP.

Limitations of this study include the small sample size, which needs to be prospectively augmented in the future. Furthermore, the retrospective character of the analysis does not allow for identification of causative factors leading to either the elevation or decrease of ONSD. In particular, the exact influence of corticosteroids on ONSD, especially with regard to possible reduction intervals, cannot be precisely addressed.

Future applications of ONSD in patients with GBM may thus include comparison of ONSD with invasively measured ICP and volumetry of perifocal edema. In addition, it would be interesting to follow‐up patients with GBM by means of MRI‐ or US‐based ONSD and pay close attention to the influence of adjuvant radio‐chemotherapy, corticosteroid administration, and/or recurrence, ideally in a prospective setting.

## 5 CONCLUSIONS

Measuring ONSD might help identify intracranial hypertension in patients with GBM. In this first‐of‐its kind study, we observed a significant increase of ONSD from time of diagnosis to time of surgery, probably associated with edema and tumor mass effects. No significant effect of dexamethasone was observed; further investigation is necessary. Postoperatively, ONSD decreased significantly until 3 months after surgery before increasing again for unknown reasons. ONSD is a promising adjunct to clinical care to assess elevated ICP in GBM. A possible use may lie in supporting surgical decision‐making concerning timing of surgery. Also, ONSD may help assessing the effects of postoperative adjuvant therapy on ICP.

## AUTHOR CONTRIBUTIONS

**Daniel Feucht:** Conceptualization (equal); data curation (equal); formal analysis (equal); visualization (equal); writing – original draft (equal); writing – review and editing (equal). **Susanne R. Kerscher:** Conceptualization (equal); methodology (equal); supervision (equal); validation (equal); writing – review and editing (equal). **Christer Ruff:** Writing – review and editing (equal). **Martin U. Schuhmann:** Conceptualization (equal); supervision (equal); writing – review and editing (equal). **Constantin Roder:** Writing – review and editing (equal). **Julian Zipfel:** Conceptualization (equal); data curation (equal); formal analysis (equal); investigation (equal); project administration (equal); supervision (equal); visualization (equal); writing – original draft (equal); writing – review and editing (equal).

## FUNDING INFORMATION

This work was not supported by any grant. We acknowledge support from the Open Access Publication Fund of the University of Tübingen.

## CONFLICT OF INTEREST STATEMENT

The authors declare no conflicts of interest.

## ETHICS STATEMENT

Institutional review board approval was granted by the Ethics Committee of University of Tuebingen (793/2022BO2) on December 21, 2022.

## Data Availability

The data that support the findings of this study are available from the corresponding author upon reasonable request.
